# The efficacy of transcatheter arterial chemoembolization for hepatocellular carcinoma: is the alteration of the inflammation index important?

**DOI:** 10.3389/fmed.2025.1543903

**Published:** 2025-03-14

**Authors:** Chao Luo, Hua Xiang, Jie Tan

**Affiliations:** ^1^Department of Interventional Vascular Surgery, Hunan Provincial People’s Hospital (The First Affiliated Hospital of Hunan Normal University), Changsha, Hunan, China; ^2^Department of Interventional Vascular Surgery, Hunan Cancer Hospital, The Affiliated Cancer Hospital of Xiangya School of Medicine, Central South University, Changsha, Hunan, China

**Keywords:** hepatocellular carcinoma, chemoembolization, peripheral inflammation, inflammation index, prognosis

## Abstract

**Introduction:**

Transcatheter arterial chemoembolization (TACE) is widely applied for locoregional malignant lesions control in intermediate and selected advanced hepatocellular carcinoma (HCC). Various inflammation indices, such as neutrophil-to-lymphocyte ratio (NLR), lymphocyte-to-monocyte ratio (LMR), platelet-to-lymphocyte ratio (PLR), systemic immune inflammatory index (SII), and Lymphocyte-to-C Reactive Protein Ratio (LCR) have been explored as tools for predicting the efficacy of TACE. However, the role and predictive value for dynamic changes of peripheral inflammatory indicators pre- and post-TACE remains unclear.

**Objective:**

To explore the association between the alteration in inflammatory index and the efficacy and prognosis of TACE and to provide more evidence for early prediction of the efficacy of TACE.

**Methods:**

This was a retrospective single-center study. HCC patients who received TACE as initial treatment were enrolled. The relationship between the alteration of inflammation indices (calculated as post-TACE minus pre-TACE measurements) and TACE efficacy and prognosis was investigated. Progression-free survival (PFS) was the primary endpoint, and treatment efficacy was evaluated based on mRECIST criteria.

**Results:**

Before propensity score matching (PSM), the change in LMR was significantly associated with treatment effective rate, with the unelevated ΔLMR group achieving a 79.4% treatment effective rate compared to 36.4% in the elevated group (*p* < 0.001). The estimated median PFS was 9.7 months and 4.5 months in the unelevated and elevated group, with a significant difference (*p* = 0.016). After PSM, the treatment effective rate was 48.7 and 38.5% (*p* = 0.214), and the estimated median PFS was 8.9 and 5.5 months (*p* = 0.173) for the unelevated and elevated group, respectively.

**Conclusion:**

Our study demonstrated that alteration of indices of peripheral inflammation, including ΔNLR, ΔLMR, ΔSII, and ΔPLR, may not be valuable prognostic markers for HCC patients who received TACE.

## Introduction

Primary liver cancer is the sixth most common cancer in terms of new cases each year and the third most common cause of cancer-related deaths worldwide, with more than 900,000 new cases and more than 830,000 deaths each year (1). Hepatocellular carcinoma (HCC) represents 75–90% of primary liver cancer (2, 3). Standard treatments for HCC include liver transplantation, surgical resection, local treatments such as transcatheter arterial chemoembolization (TACE), transcatheter arterial radioembolization (TARE), ablation, and systemic therapies (4). According to the Barcelona Clinic for Liver Cancer (BCLC) staging guideline, TACE is recommended for intermediate HCC, and there is growing evidence supporting its use in selected cases of advanced HCC (5, 6).

To date, various inflammation indices, such as neutrophil-to-lymphocyte ratio (NLR), lymphocyte-to-monocyte ratio (LMR), platelet-to-lymphocyte ratio (PLR), systemic immune inflammatory index (SII), and lymphocyte-to-C reactive protein ratio (LCR) have been explored as tools for predicting the efficacy of TACE (7–9). However, the predictive value of dynamic changes in these peripheral inflammatory indicators pre- and post-TACE remains unclear. Therefore, this retrospective study aims to assess the association between the alteration in inflammatory index and TACE efficacy and prognosis, providing further evidence for early prediction of treatment outcome.

## Materials and methods

### Study population

**Figure 1:**
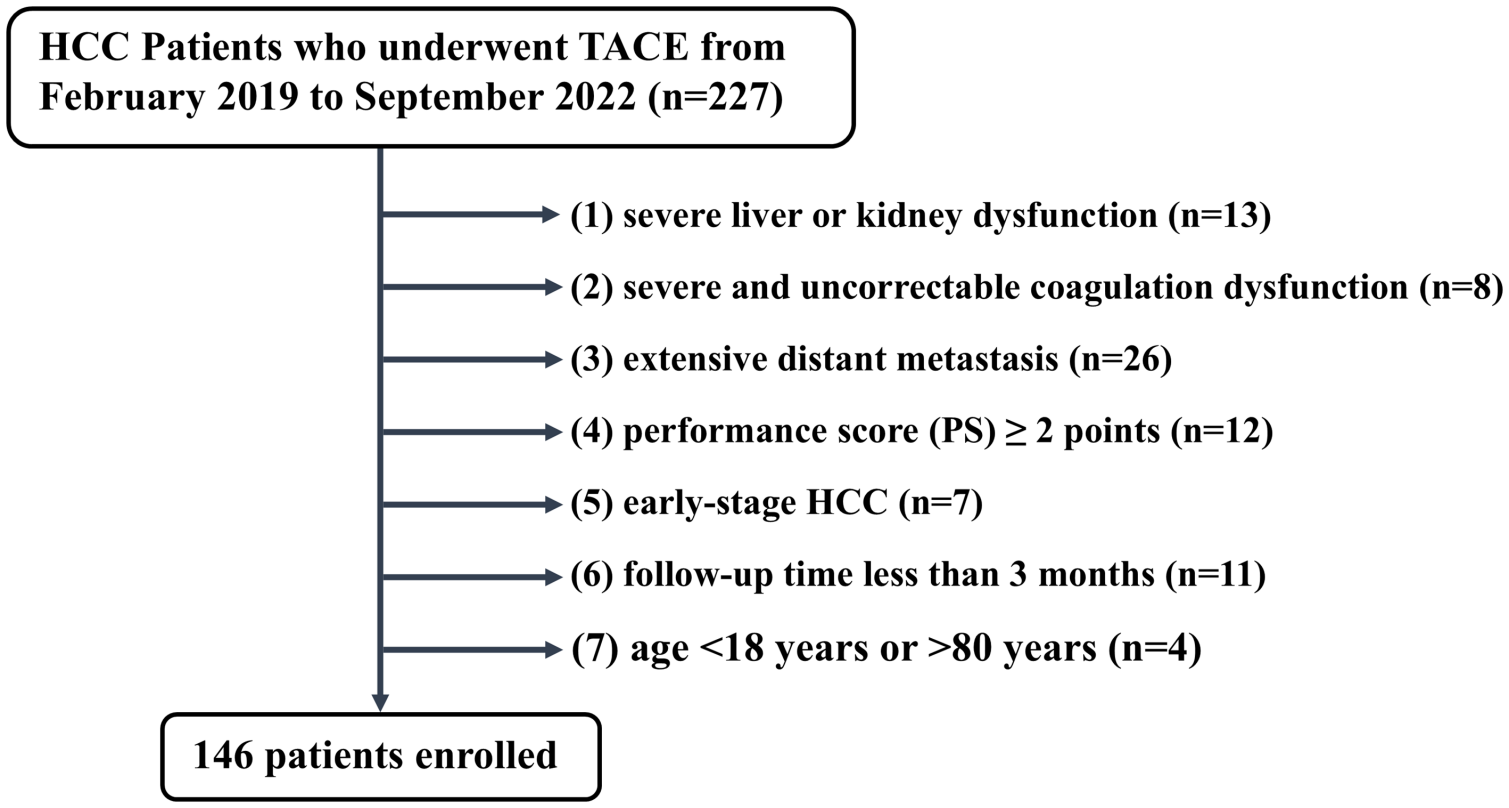
The flowchart of the studied population.

This single-center, retrospective study was approved by the ethics committee of Hunan Provincial People’s Hospital and was performed following the Declaration of Helsinki (10). The requirement for written informed consent was waived by the institutional review boards due to the retrospective nature of this study. A total of 227 HCC patients who received TACE as initial treatment at Hunan Provincial People’s Hospital (First Affiliated Hospital of Hunan Normal University) between February 2019 and September 2022 were included. Diagnosis of HCC was confirmed using the Liver Imaging Reporting and Data System (Li-RADS) or pathological confirmation (11). Exclusion criteria were: (1) severe liver or kidney dysfunction; (2) severe and uncorrectable coagulation dysfunction; (3) extensive distant metastasis; (4) performance score (PS) ≥ 2; (5) early-stage HCC; (6) follow-up time of <3 months after the initial TACE; (7) age < 18 or > 80 years. The final cohort included 146 patients, as illustrated in Figure 1.

### Data collection and definition

Clinical and demographic data were collected from the electronic medical records, including age, gender, hepatitis B surface antigen (HBsAg) status, alpha-fetoprotein (AFP) levels, alkaline phosphatase (ALP), BCLC stage, Child-Pugh score, initial TACE treatment response, and complete blood counts (neutrophil count, lymphocyte count, monocyte count, and platelet count). Inflammatory indices were calculated as follows: NLR = neutrophil count/lymphocyte count; LMR = lymphocyte count/monocyte count; SII = (neutrophil × platelet)/lymphocyte count; PLR = platelet count/lymphocyte count. Indices were reexamined 3 days post-TACE. The alteration was represented as delta (Δ) value, calculated by subtracting the pre-TACE value from the post-TACE value. Positive ΔNLR, ΔLMR, ΔSII, and ΔPLR indicated elevation, while negative and null values indicated no elevation.

### TACE procedure

All patients were reviewed by a multidisciplinary tumor board. TACE is recommended for patients who are not suitable for or refuse curative treatment. Procedures were conducted by board-certified interventional radiologists using femoral or radial artery access. For femoral access, 5-Fr Yashiro or Rosch hepatic catheters were used, while vertebral or multipurpose catheters were employed via radial access. Angiography of the celiac and superior mesenteric arteries identified intrahepatic tumors and their blood supply. Microcatheters (2.7-F or 2.8-F) enabled superselective embolization based on angiographic findings.

In conventional TACE (cTACE), an iodized oil-idarubicin/doxorubicin emulsion was prepared with up to 15 mL of lipiodol and 10 mg idarubicin or 40–80 mg doxorubicin, with a 3:1 or 2:1 volume ratio of iodized oil to aqueous solvent (either sugar solution or sterile water for injection). This mixture follows the “water-in-oil” technique commonly used in cTACE (12). Embolization of proximal tumor feeders was achieved using Gelfoam slurry, microspheres (Embozene, Varian Medical Systems), or polyvinyl alcohol (PVA) particles (Cook Medical). In cases with hepatic arteriovenous fistulas, PVA particles or microspheres were applied before lipiodol embolization.

For drug-eluting bead TACE (DEB-TACE), beads (Biocompatibles, UK; Jiangsu Hengrui Medical, China) were loaded with idarubicin (10 mg) or doxorubicin (40–80 mg) for 20–30 min, and then delivered into the tumor’s feeding artery based on angiographic evaluation. All procedures adhered to the Society of Interventional Radiology (SIR) guidelines (13).

### Follow-up and outcome

Patients underwent imaging follow-ups, including hepatic contrast-enhanced CT/MRI and chest CT, every 1–3 months post-TACE. Repeated TACE treatments were administrated as needed, based on active tumor remnant and liver function. The treatment effect of target lesions and non-target lesions and the presence of new lesions are comprehensively evaluated. The overall efficacy, according to the mRECIST criteria, was divided into complete remission (CR), partial remission (PR), stable disease (SD), and progressive disease (PD) (14). CR and PR were defined as treatment effective, while SD and PR were treatment ineffective. The primary endpoint was progression-free survival (PFS), defined as the time from the initial TACE to disease progression, death, or the last follow-up. Overall survival (OS) was the second endpoint, defined as the time from the initial TACE and death or the last follow-up. The last follow-up ended on October 21, 2023. Censored data was recorded if the endpoint event had not been reached at the last follow-up.

### Statistical methods

Continuous variables were expressed as mean ± standard deviation (SD) or median and interquartile range (IQR). Categorical variables were presented as counts and percentages. Comparisons were made using the *χ*^2^ test or Fisher’s exact test, as appropriate. Logistic regression analysis identified risk factors for ineffective initial TACE response, while Cox proportional hazards models determined independent risk factors for PFS and OS. Variables with *p* < 0.1 in the univariate analyses were included in the multivariate analyses. To mitigate potential confounding and selection bias between the two groups, propensity score matching (PSM) was employed. A 1:1 nearest-neighbor matching without replacement was performed using a caliper width of 0.1 standard deviations of the logit of the propensity score (15). The propensity score was estimated using a logistic regression model, adjusting for the covariates listed in Table 1. The caliper width of 0.1 ensures that matches are restricted to within 0.1 standard deviations of the propensity score, improving the balance between groups while minimizing bias. The Kaplan–Meier method was used to estimate PFS and OS between different groups, and survival curves were generated to compare different groups. Differences between survival curves were assessed using the log-rank test. Kaplan–Meier survival analysis was performed using the *survival* package and visualized with *survminer* package in R software (16, 17). A *p* < 0.05 was considered statistically significant. Analyses were conducted using statistical software (SPSS version 27, International Business Machines Corporation) and R software (version 4.0.2).^1^

**Table 1:** Baseline characteristics of unelevated and elevated LMR groups before and after PSM.

Characteristics	Before PSM			After PSM		
	Unelevated LMR group (*n* = 102)	Elevated LMR group (*n* = 44)	*P*	Unelevated LMR group (*n* = 39)	Elevated LMR group (*n* = 39)	*P*
Gender			0.308			0.834
Male	86 (84.3)	34 (77.3)		29 (74.4)	31 (79.5)	
Female	16 (15.7)	10 (22.7)		10 (25.6)	8 (20.5)	
Age			0.828			1.000
<60	53 (52.0)	22 (50.0)		21 (53.8)	21 (53.8)	
≥60	49 (48.0)	22 (50.0)		18 (46.2)	18 (46.2)	
BCLC stage			0.001			0.138
B	53 (52.0)	15 (34.1)		11 (28.2)	15 (38.5)	
C	49 (48.0)	29 (65.9)		28 (71.8)	24 (61.5)	
HBsAg			0.042			0.714
Positive	80 (78.4)	38 (86.4)		30 (76.9)	33 (84.6)	
Negative	22 (21.6)	6 (13.6)		9 (23.1)	6 (15.4)	
Cirrhosis			0.265			0.928
Present	62 (60.8)	31 (70.5)		25 (64.1)	26 (66.7)	
Absent	40 (39.2)	13 (29.5)		14 (35.9)	13 (33.3)	
AFP (ng/mL)			0.021			0.641
<400	60 (58.8)	23 (52.3)		24 (61.5)	22 (56.4)	
≥400	42 (41.2)	21 (47.7)		15 (38.5)	17 (43.6)	
Child-Pugh grade			0.184			0.797
A	77 (75.5)	29 (65.9)		25 (64.1)	27 (69.2)	
B	25 (24.5)	15 (34.1)		14 (35.9)	12 (30.8)	
Type of TACE			0.950			1.000
cTACE	25 (24.5)	11 (25.0)		10 (25.6)	10 (25.6)	
DEB-TACE	77 (75.5)	33 (75.0)		29 (74.4)	29 (74.4)	
Pre-TACE LMR	3.10 ± 2.13	2.11 ± 1.12	0.004	2.62 ± 1.83	2.28 ± 1.05	0.286
Treatment efficacy of the initial TACE			<0.001			0.214
Effective	81 (79.4)	16 (36.4)		19 (48.7)	15 (38.5)	
Ineffective	21 (20.6)	28 (63.6)		20 (51.3)	24 (61.5)	

LMR, Lymphocyte to monocyte ratio; PSM, propensity score matching, BCLC, Barcelona Clinic Liver Cancer; HBsAg, Hepatitis B surface Antigen; AFP, alpha-fetoprotein; TACE, Transcatheter arterial chemoembolization; the number in parentheses represents the percentage.

## Results

### Clinical baseline characteristics

Among the 146 HCC patients who received TACE as initial treatment, 120 were male, and 26 were female. The median age was 59 years, with 75 patients younger than 60. Sixty-eight patients were classified as BCLC stage B (intermediate), while 78 patients were classified as stage C (advanced). A total of 106 patients tested positive for hepatitis B surface antigen (HBsAg), and 93 patients had cirrhosis. Forty patients had a Child-Pugh grade B classification, and 63 patients (42%) presented with AFP levels ≥400 ng/mL.

Regarding inflammatory indices, 18 patients (12%) exhibited decreased NLR, 102 patients (70%) showed decreased LMR, 25 patients (17%) had decreased SII, and 35 patients (24%) showed decreased PLR. The median follow-up period was 18.6 months. Detailed baseline characteristics are provided in Table 2.

**Table 2:** The baseline characteristics of all patients included in analysis.

Characteristics	Overall (*n* = 146, %)	Characteristics	Overall (*n* = 146, %)
Gender		Type of TACE	
Male	120 (82.2)	cTACE	36 (24.7)
Female	26 (17.8)	DEB-TACE	110 (75.2)
Age		ΔNLR	
<60	75 (51.4)	Unelevated	18 (12.3)
≥60	71 (48.6)	Elevated	128 (87.7)
BCLC stage		ΔLMR	
B	68 (46.6)	Unelevated	102 (69.9)
C	78 (53.4)	Elevated	44 (30.1)
HBsAg		ΔSII	
Positive	106 (72.6)	Unelevated	25 (17.1)
Negative	40 (27.4)	Elevated	121 (82.9)
Cirrhosis		ΔPLR	
Present	93 (63.7)	Unelevated	35 (24.0)
Absent	53 (36.3)	Elevated	111 (76.0)
AFP (ng/mL)		Response to the initial TACE	
<400	83 (56.8)	CR	19 (13.0)
≥400	63 (43.2)	PR	78 (53.4)
Child-Pugh grade		SD	3 (2.1)
A	106 (72.6)	PD	46 (31.5)
B	40 (27.4)		

BCLC, Barcelona Clinic Liver Cancer; HBsAg, Hepatitis B surface Antigen; AFP, alpha-fetoprotein; TACE, Transcatheter arterial chemoembolization; NLR, Neutrophil to lymphocyte ratio; LMR, Lymphocyte to monocyte ratio; SII, Systemic inflammatory index; PLR, Platelet to lymphocyte ratio; AFP, Alpha fetoprotein; CR, Complete response; PR, Partial response; SD, Stable disease; PD, Progressive disease.

### Logistic regression analysis of ineffective response for the initial TACE

In univariate logistic regression analysis, ineffective response for the initial TACE was significantly associated with BCLC stage, serum AFP level, and the alteration of LMR. Multivariate analysis identified BCLC stage C [Odds ratio (OR): 3.475; 95% confidence interval (CI): 1.417–8.526], AFP ≥ 400 ng/mL (OR: 3.114; 95% CI: 1.333–7.275), and elevated LMR (OR: 8.585; 95% CI: 3.538–20.832) as independent risk factors for ineffective treatment. Specific results are detailed in Table 3.

**Table 3:** Uni- and multivariate logistic regression analysis of the risk factors of ineffective response for the initial TACE.

Variable	Univariate analysis		Multivariate analysis	
	OR (95% CI)	*P*-value	OR (95% CI)	*P*-value
Gender		0.560		
Male	Reference			
Female	0.770 (0.320–1.853)			
Age		0.092		0.428
<60	Reference		Reference	
≥60	0.548 (0.272–1.103)		0.737 (0.347–1.567)	
BCLC stage		0.001		**0.007**
B	Reference		Reference	
C	3.626 (1.712–7.682)		3.475 (1.417–8.526)	
HBsAg		0.821		
Negative	Reference			
Positive	1.092 (0.508–2.349)			
Cirrhosis		0.774		
Absent	Reference			
Present	1.111 (0.542–2.278)			
AFP (ng/mL)		0.001		**0.009**
<400	Reference		Reference	
≥400	3.498 (1.705–7.177)		3.114 (1.333–7.275)	
Child-Pugh grade		0.821		
A	Reference			
B	1.092 (0.508–2.349)			
Type of TACE		0.660		
cTACE	Reference			
DEB-TACE	1.199 (0.533–2.698)			
ΔNLR		0.983		
Unelevated	Reference			
Elevated	1.012 (0.355–2.881)			
ΔLMR		<0.001		**<0.001**
Unelevated	Reference		Reference	
Elevated	6.750 (3.096–14.718)		8.585 (3.538–20.832)	
ΔSII		0.759		
Unelevated	Reference			
Elevated	1.136 (0.503–2.564)			
ΔPLR		0.777		
Unelevated	Reference			
Elevated	0.878 (0.357–2.160)			

OR, Odds ratio; 95% CI, 95% confidence interval; BCLC, Barcelona Clinic Liver Cancer; HBsAg, Hepatitis B surface Antigen; AFP, alpha-fetoprotein; TACE, Transcatheter arterial chemoembolization; NLR, Neutrophil to lymphocyte ratio; LMR, Lymphocyte to monocyte ratio; SII, Systemic inflammatory index; PLR, Platelet to lymphocyte ratio. Bold values in indicate independent risk factors identified through multivariate regression analysis.

### Cox regression analysis for PFS and OS

In Cox univariate regression analysis, BCLC stage, AFP level, alteration of LMR, and initial TACE response significantly influenced PFS. Multivariate analysis identified BCLC stage C [Hazard Ratio (HR): 1.651; 95% CI: 1.040–2.619], AFP ≥ 400 ng/mL (HR: 1.662; 95% CI: 1.060–2.608), and initial TACE treatment ineffective (HR: 2.116; 95% CI: 1.327–3.376) as independent risk factors for disease progression. Table 4 presents the detailed result.

**Table 4:** Uni- and multivariate Cox regression analysis of the risk factors associated with lower PFS after the initial TACE.

Variable	Univariate analysis		Multivariate analysis	
	HR (95% CI)	*P*-value	HR (95% CI)	*P*-value
Gender		0.131	–	–
Male	Reference			
Female	1.466 (0.892–2.411)			
Age		0.315		
<60	Reference			
≥60	0.815 (0.548–1.214)			
BCLC stage		<0.001		**0.033**
B	Reference		Reference	
C	2.303 (1.523–3.482)		1.651 (1.040–2.619)	
HBsAg		0.632		
Positive	Reference			
Negative	1.116 (0.713–1.747)			
Cirrhosis		0.911		
Present	Reference			
Absent	0.977 (0.644–1.481)			
AFP (ng/mL)		<0.001		
<400	Reference		Reference	**0.027**
≥400	2.419 (1.619–3.613)		1.662 (1.060–2.608)	
Child-Pugh grade		0.577		
A	Reference			
B	1.135 (0.727–1.774)			
Type of TACE		0.062		0.171
cTACE	Reference		Reference	
DEB-TACE	1.585 (0.977–2.572)		1.411 (0.862–2.309)	
ΔNLR		0.286		
Unelevated	Reference			
Elevated	0.734 (0.415–1.296)			
ΔLMR		0.017		0.466
Unelevated	Reference		Reference	
Elevated	1.655 (1.094–2.504)		1.189 (0.747–1.891)	
ΔSII		0.171		
Unelevated	Reference			
Elevated	0.707 (0.430–1.162)			
ΔPLR		0.183		
Unelevated	Reference			
Elevated	0.733 (0.465–1.157)			
Response of the initial TACE		<0.001		**0.002**
Effective	Reference		Reference	
Ineffective	2.729 (1.829–4.072)		2.116 (1.327–3.376)	

PFS, progression free survival; TACE, Transcatheter arterial chemoembolization; HR, Hazard Ratio; 95% CI, 95% confidence interval; BCLC, Barcelona Clinic Liver Cancer; HBsAg, Hepatitis B surface Antigen; AFP, alpha-fetoprotein; NLR, Neutrophil to lymphocyte ratio; LMR, Lymphocyte to monocyte ratio; SII, Systemic inflammatory index; PLR, Platelet to lymphocyte ratio. Bold values indicate independent risk factors identified through multivariate regression analysis.

For OS, univariate regression analysis revealed a significant association with BCLC stage, AFP level, and response of the initial TACE. Multivariate analysis showed that BCLC stage C (HR: 2.473; 95% CI: 1.534–3.987), Child-Pugh grade B (HR: 1.759; 95% CI: 1.117–2.770), and the ineffective initial TACE treatment (HR: 1.802; 95% CI: 1.153–2.818) were independent risk factors for mortality. Detailed results are shown in Table 5.

**Table 5:** Uni- and multivariate Cox regression analysis of the risk factors associated with lower OS after the initial TACE.

Variable	Univariate analysis		Multivariate analysis	
	HR (95% CI)	*P*-value	HR (95% CI)	*P*-value
Gender		0.313		
Male	Reference			
Female	1.323 (0.768–2.281)			
Age		0.113		
<60	Reference			
≥60	0.714 (0.470–1.083)			
BCLC stage		<0.001		**<0.001**
B	Reference		Reference	
C	2.705 (1.746–4.189)		2.473 (1.534–3.987)	
HBsAg		0.863		
Positive	Reference			
Negative	0.960 (0.606–1.522)			
Cirrhosis		0.771		
Present	Reference			
Absent	0.938 (0.610–1.443)			
AFP (ng/mL)		0.001		0.357
<400	Reference		Reference	
≥400	2.019 (1.335–3.053)		1.246 (0.780–1.991)	
Child-Pugh grade		0.063		**0.015**
A	Reference		Reference	
B	1.528 (0.977–2.391)		1.759 (1.117–2.770)	
Type of TACE		0.189		
cTACE	Reference			
DEB-TACE	1.404 (0.846–2.330)			
ΔNLR		0.780		
Unelevated	Reference			
Elevated	0.913 (0.485–1.722)			
ΔLMR		0.495		
Unelevated	Reference			
Elevated	1.170 (0.746–1.834)			
ΔSII		0.307		
Unelevated	Reference			
Elevated	0.762 (0.453–1.282)			
ΔPLR		0.324		
Unelevated	Reference			
Elevated	0.788 (0.490–1.266)			
Response of the initial TACE		0.001		**0.010**
Effective	Reference		Reference	
Ineffective	2.081 (1.366–3.171)		1.802 (1.153–2.818)	

OS, overall survival; TACE, Transcatheter arterial chemoembolization; HR, Hazard Ratio; 95% CI, 95% confidence interval; BCLC, Barcelona Clinic Liver Cancer; HBsAg, Hepatitis B surface Antigen; AFP, alpha-fetoprotein; NLR, Neutrophil to lymphocyte ratio; LMR, Lymphocyte to monocyte ratio; SII, Systemic inflammatory index; PLR, Platelet to lymphocyte ratio. Bold values indicate independent risk factors identified through multivariate regression analysis.

### Comparison of TACE efficacy, PFS, and OS between ΔLMR groups before and after PSM

Before PSM, 102 patients were in the unelevated LMR group, and 44 patients were in the elevated group. The unelevated LMR group had a significantly lower rate of BCLC stage C (*p* = 0.001), positive HBsAg (*p* = 0.042), and AFP ≥400 ng/mL (*p* = 0.021), along with higher pre-TACE LMR (*p* = 0.004). Treatment efficacy was 79.4% in the unelevated group compared to 36.4% in the elevated group (*p* < 0.001).

**Figure 2:**
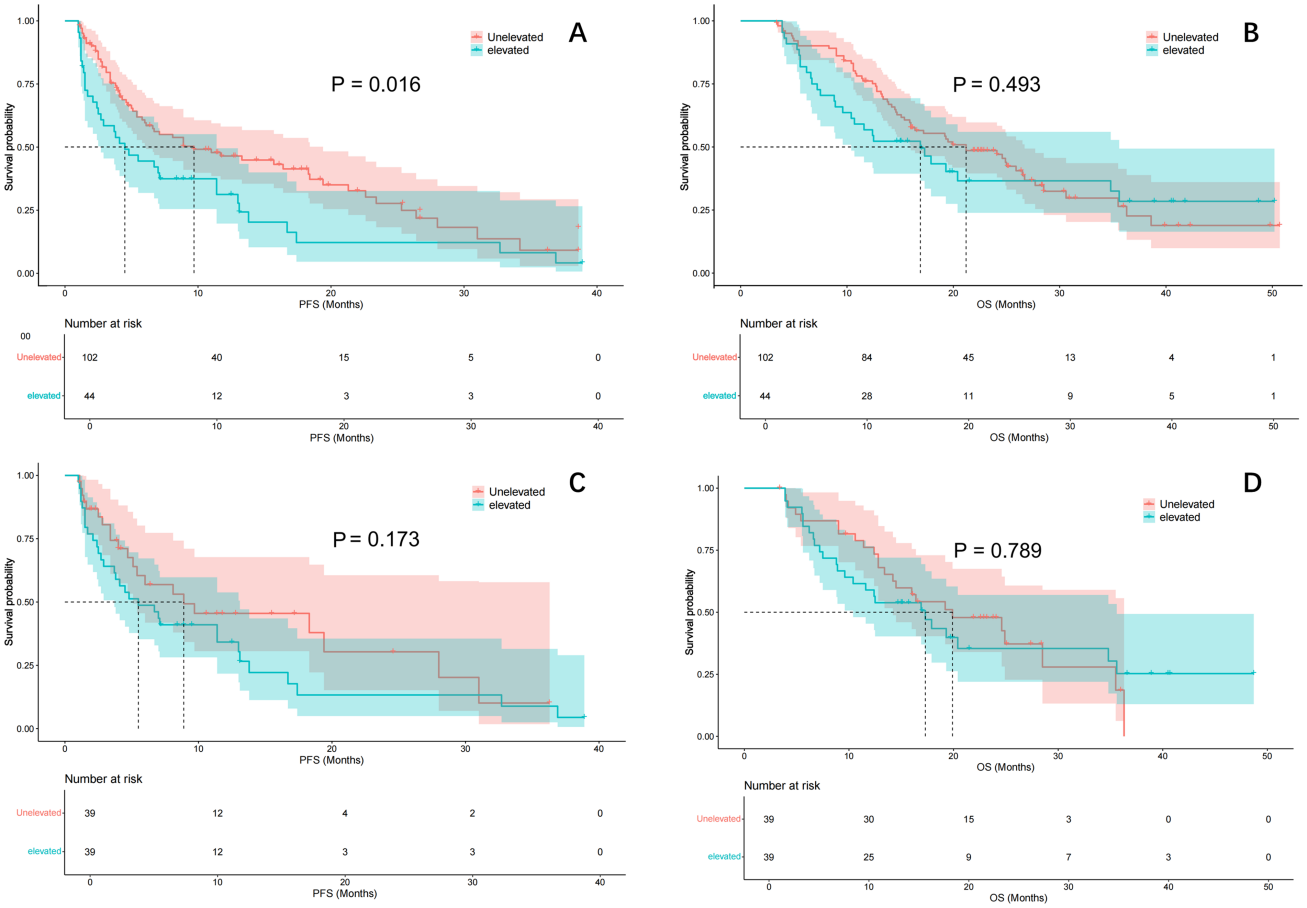
Comparison of PFS and OS curve between ΔLMR group before and after PSM. Kaplan–Meier curve comparison of patients with unelevated and elevated LMR group: **(A)** PFS; **(B)** OS; **(C)** PFS after adjustment by PSM; **(D)** OS after adjustment by PSM. PFS, progression-free survival; OS, overall survival; LMR, lymphocyte to monocyte ratio; PSM, propensity score matching.

After one-to-one PSM analysis, 39 patient pairs were matched, and baseline characteristics showed no significant differences (Table 1). The estimated median PFS was 9.7 months in the unelevated LMR group and 4.5 months in the elevated group before PSM (*p* = 0.016) (Figure 2A). No significant difference in OS was observed between the groups (*p* = 0.493), with estimated median OS of 21.2 and 16.9 months, respectively (Figure 2B).

Post-PSM analysis showed a treatment efficacy of 48.7% in the unelevated LMR group and 38.5% in the elevated group (*p* = 0.214). The estimated median PFS was 8.9 and 5.5 months in the unelevated and elevated groups, respectively (*p* = 0.173) (Figure 2C). The estimated median OS was 19.9 and 17.3 months in the unelevated and elevated groups, respectively (*p* = 0.789) (Figure 2D).

## Discussion

Our study evaluated the prognostic significance of changes in peripheral inflammatory indices pre- and post-TACE in HCC patients. Our findings indicate that alterations in NLR, SII, and PLR are not significantly associated with TACE efficacy or prognosis. However, elevated LMR was linked to poorer treatment response of the initial TACE and inferior PFS before PSM, although this association diminished after adjustment. The role of alteration of peripheral inflammatory index pre- and post-TACE is seldom studied, but several preoperative inflammatory markers have been explored as predictive biomarkers in HCC. The peripheral inflammatory index could reflect the interaction between the tumor biology, the tumor stromal microenvironment (TME), and the immune response (18). Thus, it possesses the capability to predict treatment response, recurrence, progression, and overall survival of TACE (19, 20). A retrospective study conducted by Minic et al. (8) divided pre-TACE LMR into the low and high groups by 2.24, and the high LMR group exhibited superior TACE efficacy and better PFS. Similarly, Yang et al. (21) reported that high preoperative LMR predicted poor OS and disease-free survival (DFS) in HCC patients undergoing hepatectomy, while NLR and PLR did not.

Our study found that the elevated LMR group presented with significantly lower pre-TACE LMR levels, more advanced BCLC stage, HBsAg positivity, and elevated AFP levels. However, after PSM, the previously observed advantage of unelevated LMR on treatment response and PFS was no longer significant. This suggests that the observed associations may be confounded by baseline patient characteristics rather than the alteration in LMR itself. LMR is calculated by both the count of lymphocytes and monocytes in the peripheral blood. Lymphocytes are a type of inflammatory cell that is also responsible for anti-tumor immunity. The participation of lymphocytes (such as T cells) in tumor infiltration is generally associated with improved prognosis in cancer patients and has been applied for targeted therapy (22). Lymphopenia may indicate abnormal immune mechanisms and a decrease in immune surveillance to remove tumor cells that may affect the tumor microenvironment (23). Thus, the decrease in circulating lymphocytes is detrimental to tumor control. As for monocytes, those cells migrate to liver cancer tissues in the blood circulation and differentiate into macrophages and dendritic cells (DCs), demonstrating both pro-tumor and anti-tumor effects. Monocytes can produce tumor-killing mediators and stimulate NK cells (24). However, in the tumor microenvironment (TME), monocytes also promote immunosuppression, extracellular matrix (ECM) remodeling, angiogenesis, and cancer cell intravasation. In addition, monocytes can differentiate into tumor-associated monocytes (TAMs) that support tumor growth (25). All in all, lower LMR is associated with a rough intrahepatic immune microenvironment, thus leading to a more malignant characteristic, resistance to locoregional therapy, and being more prone to progression.

Interestingly, TACE-induced tumor embolization may also modulate the intrahepatic immune response. Animal research by Tischfield et al. (26) discovered that embolization of the HCC lesions could regulate intrahepatic lymphocytes, with upregulated CD4+ cells and downregulated CD8+ cells, and a reduction in the proportion of CD25+/CD4 + cells within the liver and target lesions. Embolization could also recruit tumor-infiltrated lymphocytes (TIL) and modulate PD-L1 expression. However, the relationship between intrahepatic immune changes and peripheral inflammatory indices remains unclear. Investigating the immune cell composition within tumors and correlating these findings with circulating biomarkers such as cytokines or microRNAs may provide deeper insights into treatment response and disease progression in HCC. Combined with our findings, peripheral inflammatory responses may not reliably predict TACE outcomes due to the complexity of immune modulation within the TME (27).

Our study also identified BCLC stage C, AFP ≥ 400 ng/mL, and elevated LMR as independent risk factors for ineffective initial TACE treatment. In the Cox regression analysis of PFS and OS, BCLC stage C, AFP ≥ 400 ng/mL, and initial TACE treatment ineffective were independent risk factors for disease progression, while BCLC stage C, Child-Pugh grade B, and initial TACE treatment ineffective were independent risk factors for death after the initial TACE. These findings are consistent with previous studies highlighting the prognostic importance of tumor burden and treatment response (3, 4). A retrospective study by Kim et al. (28) concluded that achievement of objective response, in particular CR, at the initial TACE is still the robust prognostic predictor. Moreover, the concept of TACE refractory, defined as non-response after multiple TACE sessions, is receiving more attention, and patients exhibiting this pattern should be promptly considered for switch to systemic therapy (29).

There were several limitations in our study. The single-center, retrospective design and relatively small sample size may limit generalizability and introduce selection bias. Although PSM was applied to mitigate potential confounding and bias, residual heterogeneities between groups may persist. In addition, this small study conducted by PSM has some risk of overmatching. Furthermore, factors influencing inflammatory responses, such as cirrhosis, embolization extent, and infections, were not fully controlled. Another limitation is the lack of data on body mass index (BMI), which may influence inflammatory responses in cancer. Evidence suggests that underweight status may interact with inflammatory markers in gastrointestinal cancer, while overweight status in HBV-associated liver cancer has been associated with reduced PFS after TACE and persistently elevated peripheral inflammatory markers (30, 31). Future studies should explore the role of BMI in these associations to provide a more comprehensive understanding. Further multicenter, larger, prospective randomized studies are warranted to validate our findings.

## Conclusion

TACE remains a widely used treatment for intermediate and selected advanced HCC patients, with variable predicted treatment efficacy and prognosis. Our study demonstrates that alterations in peripheral inflammatory indices, including ΔNLR, ΔLMR, ΔSII, and ΔPLR, may not serve as reliable prognostic markers in this population. Future research should focus on the intrahepatic or intratumoral microenvironment and its modulation by TACE to better understand the complex interplay between the immune system and treatment outcomes.

## Data Availability

The raw data supporting the conclusions of this article will be made available by the authors, without undue reservation.

## Footnotes

^1^ http://www.R-project.org
